# Optimizing the cryopreservation of murine splenocytes for improved antigen-specific T cell function in ELISPOT

**DOI:** 10.1186/2051-1426-1-S1-P211

**Published:** 2013-11-07

**Authors:** Ekram Gad, Lauren Rastetter, Dan Herendeen, Benjamin Curtis, Meredith Slota, Marlese Koehniein, Nora Disis

**Affiliations:** 1 Oncology, University of Washington, Seattle, WA, USA

## 

ELISPOT assays are routinely used to measure immune responses of T cells in fresh and frozen splenocytes preparations; however, standardized methods for murine ELISPOT are not widely available. Freezing cells can significantly impact the function of T cells. The aim of this study was to optimize cryopreservation protocols to retain antigen-specific T cell function at similar levels as freshly isolated T cells using murine splenocytes. We examined four factors that might have an impact on cell viability and function: freezing medium, resting cells prior to freezing, temperature of the medium for initial dilution of cells after thawing and resting of cells prior to ELISPOT. FVB/N mice were vaccinated with adjuvant only (CFA/IFA) or IGF-IR peptides, previously shown to be immunogenic by ELISPOT analysis. Mouse splenocytes were cryopreserved using five different media: Medium 1 (50% X-Vivo media, 40% FBS, 10% DMSO), Medium 2 (25% RMPI, 65% FBS, 10% DMSO), Medium 3 (90% FBS, 10% DMSO), Medium 4 (Amresco Media) and Medium 5 (EZ-CP2 Media). Prior to freezing, the cells were either rested on ice for 5 hours or immediately frozen and moved to liquid nitrogen. After four weeks, we used media at 37°C or 4°C for initial dilution of cells after thawing. The thawed cells were either rested overnight at 37°C or not rested. The recovery efficiency and T cell function were evaluated by determining cell viability, background levels, peptide responses, and mitogen responses in ELISPOT assays, respectively. We observed significantly lower cell viability when using freezing Medium 4 and 5, compared to the other media tested (Medium 5 vs Media 1-4: P<0.001; Medium 4 vs 1: P<0.001, Medium 4 vs 2 P<0.05). Resting cells for 5 hours prior to freezing resulted in higher viability, however this difference was not significant (P=0.072). Using media warmed to 37°C to dilute thawed samples resulted in significantly higher cell viability as compared to using media at 4°C (P<0.0001). Media 2 and 3 gave significantly lower background than Fresh cells (P<0.05). Medium 3 was the only freezing medium to result in similar levels of IGF1R peptide responses compared to Fresh. Overnight resting of cryopreserved cells before ELISPOT gave significantly lower T cells responses in both PHA and PMA/ionomycin controls compared to unrested cryopreserved cells (P<0.001).However, overnight resting of cells gave mitogen responses most similar to that of fresh cells when PHA or PMA/ionomycin controls were used. The method of cryopreservation can have a tremendous impact upon splenocytes viability and function. By using an optimized cryopreservation protocol, it is possible to obtain antigen-specific T cell function at levels similar to freshly isolated cells.

